# Classification of overweight/obesity among Saudi adolescents relative to lifestyle behaviors using the IOTF or WHO reference standards

**DOI:** 10.3389/fped.2025.1607811

**Published:** 2025-08-11

**Authors:** Hazzaa M. Al-Hazzaa, Amal Alhakami, Ahlam M. Alotaibi

**Affiliations:** ^1^ Lifestyle and Health Research Center, Health Sciences Research Center, Princess Nourah Bint Abdulrahman University, Riyadh, Saudi Arabia; ^2^ School of Sport Sciences, University of Jordan, Amman, Jordan; ^3^ Department of Pediatrics, College of Medicine, Princess Nourah Bint Abdulrahman University, Riyadh, Saudi Arabia; ^4^ Division of Pediatric Endocrinology, Department of Pediatrics, College of Medicine, King Saud University, Riyadh, Saudi Arabia; ^5^ The University Diabetes Centre, King Saud University Medical City, King Saud University, Riyadh, Saudi Arabia

**Keywords:** BMI, adolescent, IOTF, overweight, lifestyle behaviors, obesity, underweight, WHO standards

## Abstract

**Purpose:**

To compare two BMI classifications (the IOTF and WHO references) in assessing overweight/obesity prevalence among Saudi adolescents and to evaluate the ability of the two standards to detect risks of unhealthy lifestyle behaviors.

**Methods:**

Healthy Saudi adolescents (15–19 years) were drawn from two data sets (*n* = 2,263) collected previously in 2009 and 2019, using a random multistage stratified cluster sampling technique. Measurements included weight, height, waist circumference (WC), wais to height ratio (WHtR), and selected lifestyle behaviors, including physical activity (PA), screen time, sleep duration, and dietary habits, using the Arab Teen Lifestyle Study (ATLS) questionnaire.

**Results:**

The proportions (%) of adolescents classified as underweight, normal weight, overweight, and with obesity varied according to the classification used. The IOTF system produces slightly lower overweight/obesity prevalence than the WHO standards (IOTF: 38.8%; WHO: 40.0%), with females exhibited lower overweight/obesity prevalence than males. The Kappa agreement between overweight/obesity in the two references was high (0.973), with high sensitivity (99.8%) and high specificity (98.0%). Kappa values between central obesity and both IOTF (0.691) and WHO (0.687) were moderate with moderate sensitivity and high specificity. In both classification system, intakes >4 days/week of French fries/potato chips, and chocolates/candy showed higher odds of overweight/obesity.

**Conclusion:**

Although overweight/obesity exhibited high Kappa agreement between the two classifications, variations were observed when estimating the prevalence of BMI classification using IOTF or WHO standards. Selected lifestyle behaviors showed significant associations with overweight/obesity in both classification standards.

## Background

Worldwide, childhood obesity is perhaps the most serious public health challenge, and the prevalence of pediatric obesity has considerably increased globally across different countries (1). It is well recognized that there are many major negative consequences of pediatric obesity (2, 3). Overweight and obesity in childhood and adolescence are associated with greater risk and earlier onset of chronic medical conditions, such as type 2 diabetes (2). Excess body weight in childhood and adolescence is more likely to persist in adulthood (4). Additionally, childhood and adolescence obesity have been found to have adverse psychosocial consequences and lower educational attainment (3). In a recent multi-country research, the economic burden of obesity was reported to be considerable in all the eight participating countries, regardless of geographical or economic setting, ranging from 0.8% of gross domestic product in India to as much as 2.4% in Saudi Arabia (5).

Data from the Non-Communicable Disease Risk Factor Collaboration (1) showed that obesity in children and adolescents was still more predominant than thinness among girls in 133 countries and boys in 125 countries. The increases in double burden were the consequences of upsurges in obesity (1). In Saudi Arabia, the percentage of children and adolescents classified as overweight or obese has significantly increased in the past decades (6–9). Indeed, a recent systematic review among Saudi children and adolescents reported that the ranges of overweight and obesity were greater in boys (19.3%–35.6%) than in girls (11.8%–19.2%), and that lifestyle behaviors were the most common risk factor for overweight and obesity in school-age children (8).

It is well acknowledged that the most common methods used to assess overweight or obesity among children, youth, and adults are the use of specific classification systems based on body mass index (BMI) or the use of waist circumference and waist-to-height ratio (WHtR) (1, 10–12). The implementation of school-based BMI measurement has become common as a potential method for addressing overweight and obesity among youth (13). However, defining overweight and obesity in children and adolescents is not as straightforward as it is in adults. Typically, the International Obesity Task Force (IOTF) BMI age and gender cutoff values are used to classify overweight and obesity. The classification uses a set of data collected from six countries (10). Another method is the use of the World Health Organization's (WHO) reference standards for children and adolescents aged 5–19 years, which are based on weight-for-height *Z*-scores (11). Both systems are generally valid for classifying overweight and obesity in children and adolescents. However, they often generate different findings. Within the same sample, the IOTF references tend to have the lowest prevalence (14–16). For example, a study included Saudi national data described major differences between the use of Saudi growth charts of weight for age and the WHO references (13). The study indicated that the use of the WHO standards among young Saudi children raise the prevalence of undernutrition, stunting, and wasting, which possibly results in avoidable referrals, and parental concerns (13).

Thus, it is rather challenging to determine the real prevalence of underweight, overweight, and obesity in children and adolescents when such variation exists among the most common international classification systems (10, 11). Therefore, the present study aimed to compare the two commonly BMI classification systems (the IOTF age and gender cutoff values (10), and the WHO growth references (11) that are used to evaluate overweight and obesity among children and youth, using two sets of data for Saudi adolescents between the ages of 15 and 19 years from the Arab Teens Lifestyle Study (ATLS) (17, 18). Additional objectives of the study were to determine which cutoffs for obesity agree with the criteria for central obesity using WHtR and to evaluate the potential ability of the IOTF and WHO standards to detect lifestyle-related risk factors.

## Methods

### Ethical approval

Ethical approval was obtained from the Institutional Review Board (IRB) at King Saud University, Riyadh (IRB Log Number: 17/0064/IRB) for the study conducted in 2009, and Princess Nourah Bint Abdulrahman University, Riyadh (IRB Log Number: 19-0014) for the study conducted in 2019. The research was performed in accordance with the principles stated in the Declaration of Helsinki. Written informed consent was obtained from all parents/guardians of the participating children. In addition, approval for conducting this research in schools was attained from Riyadh directorates of schools, the Ministry of Education, and the principals of the selected schools.

### Study design and participants

The present research is based on a secondary analysis of two sets of data from two studies conducted on Saudi adolescents 15–19 years of age (17, 18). The population in the two cross sectional studies was composed of healthy students of both sexes attending public and private secondary schools in Riyadh, Saudi Arabia. Riyadh city is a cosmopolitan city and the capital of Saudi Arabia. Both studies had a representative random sample size and similar design, methodology, and instrumentations. Detailed descriptions of the two studies' design and sample selection were previously published (17, 18). In brief, the required sample size was calculated assuming that the population proportion equaled 0.50, which yielded the largest possible sample size needed with a 95% confidence level and a margin of error equal to 4%. Adolescents were selected from secondary schools using a multistage stratified cluster sampling technique. Stratification was based on sex (male vs. female schools), public vs. private schools, and geographical location (east, west, north, and south). Participants were selected from the schools relative to the actual number of students in each of the public or private schools. Within each geographical location, one private and two public schools were randomly chosen. Then, classes were randomly selected from each of the three grades (10, 11, and 12). All Saudi students in the designated classes were invited to participate in the study, provided that they had no eating disorders, growth problem, or any medical condition that precluded them from engaging in physical activity.

### Anthropometric measurement and BMI classification

Measurements of weight (to the nearest 100 g) and height (to the nearest 1 cm) were conducted in the morning at the schools by trained researchers using calibrated portable scales (Seca 869, UK) and height measuring rods, respectively, while students wore minimal clothing and without shoes. Body mass index (BMI) was computed as the ratio of body weight in kilograms divided by the squared height in meters. In addition, WHtR was calculated as the ratio between WC in cm and height in cm. A WHtR cut-off point of ≥0.50 was used to define abdominal obesity in both males and females (12, 19). The WHTR is a simple, noninvasive, and practical tool that correlates well with visceral fat (12), and contrary to waist circumference, WHTR has the advantage of not requiring population specific reference tables as well as age and sex specific cutoffs (19). The ratio of 0.5 was shown to identify overweight children with the highest metabolic and cardiovascular risks (20).

Two commonly used international reference cutoff values were used to classify the BMI data. The first was the extended IOTF age- and sex-specific BMI cutoff reference standards, which are constructed based on data from adolescents in six countries: USA, UK, Brazil, the Netherlands, Hong Kong, and Singapore (10). The IOTF references provide percentile cut-offs corresponding to a BMI of 18.5, 25, and 30 kg/m^2^ at 18 years of age for underweight, overweight, and obesity status, respectively (10). The second method was the WHO growth standards for children and adolescents between the ages of 5 and 19 years, which is based on weight-for-height *Z*-scores (11). The prevalence of underweight, overweight, and obesity are defined by the WHO cut-off values as BMI-for-age less than 2 standard deviations (SDs) scores below the mean, greater than 1 SD above the mean, and greater than 2 SDs above the mean, respectively (11). The two classification systems used are based on the lambda (L), mu (M), and sigma (S) method (21). The LMS parameters correspond to median BMI (M), coefficient of variation (S), and the power in the Box–Cox transformation (L), which transforms the data, so it closely resembles a normal distribution (21). Overweight plus obesity level was calculated based on BMI ≥ 25 kg/m^2^.

### Assessment of lifestyle behaviors

Lifestyle behaviors included physical activity (PA), screen time, sleep duration, and dietary habits. They were assessed using the Arab Teen Lifestyle Study (ATLS) questionnaire (17, 22). The questionnaire has been widely used and was previously shown to be a reliable and valid instrument for assessing PA and other lifestyle habits in youth (17, 23, 24). In brief, the PA part of the questionnaire was designed to collect information on the frequency, duration and intensity of different PA during a typical week. The questionnaire covers all types of PA domains including household, transport, fitness, sporting and recreational activities. A range of diverse activities were included in the questionnaire such as walking, jogging, running, cycling, swimming, resistance training, martial arts, dancing, moderate- and vigorous-intensity sports and household PA. Time spent in all types of PA were scored in minutes per week. To classify the participants' levels of sufficient or insufficient PA, we used the WHO guidelines for PA for adolescents as reporting a total PA duration in minutes/week above or below 420 min of moderate-to-vigorous-intensity PA (25). This is equivalent to 60 min of daily PA.

The ATLS questionnaire also assesses sedentary behaviors and sleep duration. This includes questions designed to assess typical time in hours spent per day in sedentary activities (screen time), including television (TV) viewing, video games, and recreational use of computers, internet, and social media during weekdays and weekends. In addition, participants were asked to state their typical sleep duration in hours spent on weekday and weekend nights and the daily average was calculated. We used a total screen viewing time cut-off value of 3 h per day to express low and high screen time. Sufficient and insufficient sleep duration was computed as above or below 8 h per night, respectively (26). Frequency intakes of a set of dietary habits during a usual (typical) week were included in the questionnaire. They included questions about how frequently (from zero day to 7 days per week) consume breakfast, vegetables, fruits, milk/dairy products, sugar-sweetened drinks, fast foods, donuts/cakes, and sweets and chocolates. When categorizing dietary frequency, we compared daily vs. non-daily intakes of breakfast, vegetables, fruits, and milk/dairy products. Other dietary frequencies were categorized as intakes of less than 4 days per week vs. 4 plus days per week.

### Statistical analysis

Data were entered into an SPSS data file, checked for accuracy, cleaned, and analyzed using the IBM-SPSS software, version 28 (Chicago, IL, USA). Descriptive statistics were obtained for the selected variables and reported as percentages or means and standard errors. Differences between males and females in selected anthropometric and lifestyle measurements were tested using the *t*-test for independent samples. Chi-square tests of proportions were used to test the differences in BMI classifications (prevalence rates) based on the IOTF or WHO relative to underweight, normal weight, overweight, and obesity status. Agreements between BMI classification systems for detecting overweight/obesity were evaluated by Kappa measure of agreement and sensitivity and specificity were reported. We also determine which cutoff values for overweight/obesity agree with the criteria for central obesity (from WHtR data) using Kappa agreement. Finally, logistic regression analysis, adjusted for age, was used to test the associations of selected lifestyle behavioral risks with overweight/obesity vs. non-overweight/non-obesity among adolescents using each of IOTF or WHO method. The alpha level was set at ≤0.05 for significance testing.

## Results

The current study included 2,263 participants between the ages of 15 and 19 years with males representing 51.0%. The mean (SE) of age was 16.7 (0.02) years with significant differences between males and females (*p* = 0.009). Table 1 presents the anthropometric and lifestyle behaviors of the participants relative to sex. The differences between males and females were significant for all anthropometric measurements (*p* < 0.001), total physical activity (*p* = 0.005), screen time (*p* > 0.001), sleep duration (*p* > 0.001), breakfast intake (*p* > 0.001), and all other dietary habits (*p* > 0.001) except vegetable intake (*p* = 0.759).

**Table 1:** Description of anthropometric and lifestyle behaviors of the participants relative to sex.

Variable	All	Male	Female	*p*-value*
	*N* = 2,263	*N* = 1,154	*N* = 1,109	
Age	16.7 ± 0.02	16.8 ± 0.03	16.7 ± 0.03	0.009
Body weight (kg)	64.7 ± 0.42	71.1 ± 0.65	58.1 ± 0.46	<0.001
Body height (cm)	163.1 ± 0.18	169.9 ± 0.21	156.9 ± 0.17	<0.001
Body mass index (kg/m^2^)	24.2 ± 0.14	24.8 ± 0.21	23.6 ± 0.17	<0.001
Waist circumference (cm)	76.8 ± 0.33	81.1 ± 0.49	72.3 ± 0.39	<0.001
Waist to height ratio	0.47 ± 0.002	0.48 ± 0.003	0.46 ± 0.003	<0.001
Central (abdominal) obesity (%)	30.3	37.1	23.1	<0.001
Total physical activity (min/week)	406.8 ± 8.7	431.8 ± 12.9	382.4 ± 11.7	0.005
Average screen time (hours/day)	5.6 ± 0.06	5.21 ± 0.08	6.01 ± 0.09	<0.001
Average sleep time (hours/night)	7.2 ± 0.04	7.05 ± 0.05	7.34 ± 0.05	<0.001
Breakfast intake/week	3.93 ± 0.06	4.08 ± 0.08	3.78 ± 0.08	0.011
Vegetables intake/week	3.86 ± 0.06	3.84 ± 0.07	3.87 ± 0.07	0.759
Fruit intake/week	2.97 ± 0.05	3.16 ± 0.07	2.77 ± 0.07	<0.001
Milk/dairy products intake/week	4.31 ± 0.05	4.49 ± 0.07	3.13 ± 0.08	<0.001
Sweetened drinks intake/week	4.03 ± 0.05	4.24 ± 0.07	3.81 ± 0.08	<0.001
Fast food intake/week	2.87 ± 0.04	2.91 ± 0.06	2.65 ± 0.06	<0.001
French fries/potato chips intake/week	2.64 ± 0.04	2.46 ± 0.06	2.84 ± 0.06	<0.001
Cake/donuts intake/week	2.56 ± 0.05	2.38 ± 0.06	2.76 ± 0.07	<0.001
Chocolate/candy intake/week	3.48 ± 0.05	2.94 ± 0.07	4.03 ± 0.07	<0.001

Data are means ± standard error of the mean.

* *T*-test for independent samples tests for the differences between males and females.

The prevalence (%) of underweight, normal weight, overweight, and obesity among Saudi adolescents using IOTF or WHO references relative to age and sex is shown in Table 2. Generally, females' findings were significantly lower than those of males in both classifications at most of age categories. Overall, the average values of overweight/obesity using IOTF and WHO standards were 38.8% and 40.0%, respectively, with significant (*p* < 0.001) differences relative to sex. The results of Kappa agreement (not shown in the table) indicated that Kappa coefficient for the combined overweight and obesity between IOTF and WHO references was 0.973 (*p* < 0.001) with sensitivity of 99.8% and specificity of 98.0%. In addition, Kappa coefficient between central obesity and overweight/obesity by IOTF was 0.691 (*p* < 0.001) with sensitivity of 71.1% and specificity of 95.3%. On the other hand, Kappa coefficient between central obesity and combined overweight and obesity by WHO was 0.687 (*p* < 0.001) with sensitivity of 70.0% and specificity of 95.9%.

**Table 2:** The prevalence (%) of underweight, normal weight, overweight, and obesity among Saudi adolescents using IOTF or WHO reference standards relative to age groups and sex.

Age groups (years)	Reference standards^a^	Sex	Prevalence (%)				*p* value*
			Underweight	Normal weight	Overweight	Obesity	
15	IOTF	Male	9.9	43.3	22.0	24.8	0.422
		Female	8.7	50.7	23.2	17.4	
	WHO	Male	2.1	48.9	21.3	27.7	0.330
		Female	3.6	52.9	24.6	18.8	
16	IOTF	Male	10.1	44.1	21.9	23.9	0.006
		Female	9.4	55.1	21.1	14.4	
	WHO	Male	4.3	47.8	20.5	27.4	<0.001
		Female	1.2	61.4	20.8	16.7	
17	IOTF	Male	15.6	39.0	18.0	27.4	<0.001
		Female	11.8	59.2	18.8	10.2	
	WHO	Male	7.2	46.5	16.3	29.9	<0.001
		Female	2.9	67.4	19.1	10.7	
18	IOTF	Male	21.0	39.9	18.1	21.0	0.037
		Female	16.8	51.5	18.9	12.8	
	WHO	Male	9.2	51.7	14.3	24.8	<0.001
		Female	2.6	64.8	18.9	13.8	
19	IOTF	Male	22.2	44.4	15.6	17.8	0.602
		Female	16.7	37.5	29.2	16.7	
	WHO	Male	13.3	57.8	11.1	17.8	0.103
		Female	0.0^b^	54.2	29.2	16.7	
All	IOTF	Male	14.6	41.5	19.6	24.3	<0.001
		Female	11.7	54.9	20.4	13.0	
		All	13.2	48.0	20.0	18.8	
	WHO	Male	6.4	48.7	17.6	27.3	<0.001
		Female	2.3	62.9	20.5	14.3	
		All	4.4	55.6	19.0	21.0	

^a^ Overweight or obesity cut-offs are based on IOTF cut-off values (10), and WHO cut-off growth standards (11).

^b^ No participant in this cell.

* *p* values of Chi Squares tests for the differences in prevalence categories across sex for IOTF and WHO, respectively.

Table 3 displays the prevalence (%) of overweight/obesity among adolescents using IOTF or WHO reference standards relative to age groups using the two sets of data collected from the studies conducted in 2009 and 2019, separately and combined. Although there are some variations between the prevalence rates relative to ages, the findings from WHO standards exhibited significant differences relative to age categories among adolescents from data set in year 2009 (*p* = 0.045) as well as from the combined data set of 2009 and 2019 years (*p* = 0.023).

**Table 3:** The prevalence (%) of overweight/obesity among Saudi adolescents using IOTF or WHO reference standards relative to age groups and the two sets of data from studies in 2009 (reference no. 17) and 2019 (reference no. 18).

Study year	*N*	Reference standards^a^	Age groups (years)						*p* value*
			15	16	17	18	19	All	
2009	984	IOTF	42.6	40.9	32.5	38.0	45.5	38.4	0.110
	884	WHO	44.8	42.6	32.6	38.0	39.4	39.1	0.045
2019	1,244	IOTF	45.8	40.9	40.3	34.8	30.6	39.2	0.196
	1,244	WHO	49.0	42.8	41.6	35.4	30.6	40.7	0.072
Both studies	2,227	IOTF	43.7	40.9	37.1	35.7	37.7	38.8	0.147
	2,228	WHO	46.2	42.7	37.9	36.2	34.8	40.0	0.023

^a^ Overweight or obesity cut-offs are based on IOTF cut-off values (reference 10), and WHO cut-off growth standards (reference 11).

* *p* values of Chi Squares tests for the differences in prevalence categories across age groups for IOTF and WHO, respectively.

Table 4 presents the results of logistic regression analysis, adjusted for age, of selected lifestyle behaviors relative to overweight/obesity vs. non-overweight/non-obesity among participants using each of IOTF or WHO standards. Non-overweight/non-obesity was used as a reference category. There were significant negative associations (*p* < 0.001) between overweight/obesity status and increasing age when the two classification standards were used. Also, females were significantly (*p* < 0.001) less likely to have overweight, or obesity compared to males in both classification standards (IOTF: aOR = 0.647, 95% CI = 0.538–0.778; WHO: aOR = 0.658, 95% CI = 0.548–0.790). Adolescents attending public schools appeared significantly (*p* < 0.001) less likely to be overweight or have obesity compared to those in private schools. In addition, intakes ≥4 days per week of French fries/potato chips and chocolates/candy resulted in increased odds of having overweight or obesity. We also performed logistic regression analysis between those participants with obesity only vs. the other group (not shown in the table). The findings were almost like the current analysis, except that there was an additional significant (*p* = 0.033) association with breakfast intake while the associations for French fries/potato chips intake (*p* = 0.059) and chocolates/candy intake (*p* = 0.132) disappeared in the case of IOTF classification. As to WHO classification, again the association with breakfast intake became significant (*p* = 0.019), while the association with French fries/potato chips intake disappeared (*p* = 0.188).

**Table 4:** Results of logistic regression analysis, adjusted for age, of selected lifestyle behaviors relative to overweight/obesity vs. non-overweight/non-obesity among Saudi adolescents using each of IOTF or WHO.

Variable	Overweight/obesity vs. non-overweight/non-obesity^a^			
	IOTF		WHO	
	aOR (95% CI)	*p*-value	aOR (95% CI)	*p*-value
Age	0.920 (0.842–1.004)	0.062	0.885 (0.811–0.967)	0.007
Sex (male = ref)	1.00		1.00	
Female	0.647 (0.538–0.778)	<0.001	0.658 (0.548–0.790)	<0.001
School type (private = ref)	1.00		1.00	
Public	0.694 (0.567–0.849)	<0.001	0.679 (0.556–0.830)	<0.001
Physical activity (active = ref)	1.00		1.00	
Inactive (<420 min/week)	0.984 (0.812–1.192)	0.870	1.002 (0.828–1.213)	0.981
Screen time (low screen = ref)	1.00		1.00	
High screen time (>3 h)	1.029 (0.825–1.283)	0.800	1.015 (0.815–1.265)	0.893
Sleep duration (sufficient sleep = ref)	1.00		1.00	
Insufficient sleep (<8 h)	1.183 (0.984–1.422)	0.074	1.171 (0.975–1.407)	0.091
Breakfast intake (non-daily = ref)	1.00		1.00	
Daily	1.132 (0.929–1.379)	0.219	1.103 (0.907–1.343)	0.326
Vegetables intake (non-daily = ref)	1.00		1.00	
Daily	1.004 (0.801–1.258)	0.973	0.989 (0.790–1.237)	0.922
Fruit intake (non-daily = ref)	1.00		1.00	
Daily	0.909 (0.692–1.192)	0.489	0.915 (0.698–1.199)	0.519
Milk/dairy products intake (non-daily = ref)	1.00		1.00	
Daily	0.938 (0.771–1.142)	0.526	0.928 (0.763–1.128)	0.453
Sweetened drinks intake (<4 days/week = ref)	1.00		1.00	
≥4 days/week	1.014 (0.821–1.253)	0.895	0.991 (0.803–1.222)	0.931
Fast foods intake (<4 days/week = ref)	1.00		1.00	
≥4 days/week	0.936 (0.635–1.380)	0.740	0.950 (0.645–1.400)	0.796
French fries/potato chips intake (<4 days/week = ref)	1.00		1.00	
≥4 days/week	1.712 (1.137–2.580)	0.010	1.644 (1.097–2.464)	0.016
Cake/Donuts intake (<4 days/week = ref)	1.00		1.00	
≥4 days/week	0.920 (0.641–1.322)	0.653	0.979 (0.682–1.405)	0.908
Chocolates/Candy intake (<4 days/week = ref)	1.00		1.00	
≥4 days/week	1.359 (1.035–1.784)	0.027	1.377 (1.051–1.804)	0.020

^a^ Non-overweight/non-obesity was used as a reference category.

aOR, age-adjusted odds ratio; CI, confidence interval; ref, reference category; IOTF, International obesity task force age- and sex-specific BMI cutoff reference standards; WHO, World Health Organization growth references for school-aged children and adolescents.

## Discussion

The present study intended to compare the two BMI classification systems commonly used to evaluate overweight and obesity among children and youth. Namely the IOTF (10) and the WHO references (11). For this purpose, we used two sets of data for Saudi adolescents between the ages of 15 and 19 years from ATLS research (17, 18). It was also the intent of the study to determine which cutoffs for obesity agree with the criteria for central obesity, and to evaluate the potential ability of the IOTF and WHO standards to detect lifestyle-related risks. The findings showed that the average values of combined overweight and obesity using IOTF and WHO standards were 38.8% and 40.0%, respectively, with significant (*p* < 0.001) differences relative to sex.

Regardless of the BMI reference standards used, we observed a high prevalence of overweight and obesity among Saudi adolescents (IOTF = 38.8% and WHO = 40.0%), with somewhat variable but lower levels of underweight status using the WHO references. In general, compared with the results of the 2009 data set, overweight/obesity appeared somewhat higher in the results of the 2019 data sets. It is noticeable that the prevalence of overweight/obesity among Saudi adolescents has been rising over the past decades (7, 13, 18). It seems that obesity and other cardiovascular risk factors are significantly impacting on abnormal glucose metabolism in Saudi children and adolescents (27). Therefore, efforts to prevent overweight and obesity in adolescents must focus primarily on early risk identification followed by appropriate interventions.

It is rather challenging to estimate overweight and obesity prevalence when the most common international classification systems reveal different results (14–16). Overall, a higher prevalence of overweight and obesity has been reported when using WHO rather than IOTF reference values in children and adolescents from various countries across the world (14, 16, 28–33), but higher prevalence of overweight/obesity was obtained among Ukrainian children and adolescents when using IOTF classification (34). Furthermore, a school-based study conducted in eight Arab countries, including Saudi Arabia, involving adolescents aged 15–18 years reported that the use of the WHO standard yields a lower value of overweight but a higher prevalence of obesity than the use of the IOTF references (35).

Different studies have shown varying degrees of consistency when using IOTF and WHO standards for assessing overweight and obesity in children and adolescents. Among Canadian children and adolescents, the prevalence of the combined overweight/obese category is higher (35%) when based on the WHO reference compared with the IOTF standards (26%) (15). Also, a study conducted on Cree youth showed that participants classified as overweight by the IOTF system, but not by the WHO references, exhibited less severe clinical obesity (16). In other words, false-positive youth labelled with obesity status by WHO cutoffs were effectively classified as overweight by IOTF (16).

Possible explanations for the variations between IOTF and WHO methods may be attributed to the different populations, time periods, and methodologies and the approaches used to define the cutoffs used (36). It is also important to understand how the IOTF and WHO BMI standards for children and adolescents are constructed and an explanation of their inherent limitations. Adult anthropometric cutoffs are based on mortality outcomes (37). However, BMI cutoffs for children and adolescents under the age of 18 years are statistically determined (10, 11). The IOTF method uses smooth sex-specific BMI curves, constructed to match the values of 25 kg/m^2^ for overweight and 30 kg/m^2^ for obesity at 18 years. We also noticed that WHO standards underestimated “underweight” category among Saudi adolescents. This is in contrast to previous national study findings conducted on young Saudi children less than 6 years (13). This difference may be due to the fact that our sample was adolescents between 15- and 19-year-olds and the national study was conducted on children younger than 6 years. Also, our study included a sample from urban population, while the national study involved samples from rural and urban children.

In the present study, the prevalence of overweight/obesity using either IOTF or WHO standards showed higher values for males than for females. This finding aligns with those results reported in some previous studies that used the IOTF or WHO cutoff references (8). Recently, a large global study reported that a higher prevalence of overweight and obesity among school-aged boys appears more common (1). Similar results were found among a French school children (29). Other studies have reported mixed results (9, 16). However, no significant differences relative to sexes were reported among Swedish children (38). It is tempting to explain the differences in overweight and obesity between males and females shown in our study as well as in a recent local systematic review (8). The timing of pubertal onset may differently alter the body composition during adolescence. Moreover, as shown in the present study, although males are more physically active, they spend significantly more time in screen activity and have a shorter sleep duration than females. They also exhibited significantly less favorable adherence to healthy eating habits.

The Kappa coefficient between the overweight and obesity using the two reference standards was found to be high with high sensitivity and high specificity. In fact, it indicates almost perfect agreement (39). In the current study, values for Kappa agreement between central obesity and overweight and obesity using each of IOTF and WHO were considered moderate (39) along with moderate sensitivity and high specificity. Among Brazilian adolescents, a lower Kappa coefficient (0.72) was reported between the IOTF and WHO standards (28). Moreover, among French children, agreement between IOTF and WHO standards with those of French references ranged from moderate (Kappa = 0.43) to perfect (Kappa = 1.00) (29). In comparison, findings from an Italian study involving children and adolescents aged 5–17 years showed that the WHO references had the highest sensitivity, while the IOTF classification had the highest specificity when identifying subjects with obesity yet having clustered cardiometabolic risk factors (40). Moderate agreements were also observed between body fat of South American children estimated by dual-energy x-ray absorptiometry and by the IOTF (Kappa = 0.61) and WHO (Kappa = 0.63) references, with the IOTF cutoffs showing the highest specificity (0.98) (41).

The prevalence of central obesity in our study was 30.3%, with similar results for Kappa coefficient and sensitivity and specificity between central obesity and overweight and obesity using IOTF and WHO classification systems. The WHtR has gained interest as a measure of central obesity in children and adolescents (42, 43). WHtR may carry an advantage over the use of BMI alone. The WHtR association with age is weak, thus, the same cutoff value of 0.5 for central obesity has been proposed as useful across all age-groups in children (12, 42). As a result, population-specific references and age- and sex-specific cutoff values are not necessarily needed. In addition, the WHtR is considered a more reliable predictor of cardiovascular disease risk in children than BMI (43).

In the present study, we assessed the ability of the IOTF and WHO classification references to detect the association with lifestyle risk factors. Both classification systems showed significantly similar consistency of increased risks for French fries/potato chips and chocolates/Candy consumption by those adolescents with overweight or obesity. These findings agreed with the results reported by previous studies (44, 45). In fact, IOTF classification had the highest specificity in identifying participants with obesity along with clustered cardiometabolic risks (46). Also, research elsewhere showed that the means of body fat and cardiometabolic risk factors were significantly higher from normal weight to obesity, regardless of the classification system used (16).

### Strengths and limitations

The strengths of the present study include a relatively large sample size and a representative of Saudi adolescents from both public and private schools in Riyadh Additionally, BMI data came from measurements of weight and height directly and did not rely on self-report. Further, the present research is also the first to test the performance of these two reference systems against an external measure of central obesity using Saudi data. However, the present study has some limitations. First, the findings are limited to adolescents aged 15–19 years and cannot be generalized to adolescents younger than 15-year-olds. Second, the sample was drawn from an urban area and cannot be generalized to adolescents residing in rural areas, where their growth and lifestyle behaviors may be different from youth living in the cities. Third, the cross-sectional design of the present study does not imply any cause and effect between the use of BMI classification system and lifestyle risks. Fourth, we did not have data on the pubertal stages of our sample. Assessment of maturation differences within the sample and between males and females would have been useful.

## Conclusion

The IOTF classification system produces slightly lower overweight/obesity prevalence and higher rate of underweight compared with WHO references. Regardless of the classification system used, the prevalence of overweight or obese appeared to be high (IOTF = 38.8% and WHO = 40.0%). Males showed higher values than females. The Kappa coefficient of agreement for the overweight/obesity between the two references was found to be high with high sensitivity and high specificity. However, values for Kappa coefficient between central obesity (using WHtR) and overweight/obesity using each of IOTF and WHO were considered moderate along with moderate sensitivity and high specificity. In both classification systems, adolescents attending public schools were significantly less likely to be overweight or have obesity compared with adolescents attending private schools. In addition, intakes ≥4 days per week of French fries/potato chips and chocolates/candy resulted in increased odds of having overweight or obesity in both classification standards. In conclusion, despite high Kappa agreement between the two classification systems, variations were observed when estimating the prevalence of BMI classification using IOTF or WHO standards. The choice of the available BMI classification systems has important implications for adolescent's health and the assessment of clinical obesity among Saudi adolescents. Intervention to reduce overweight or obesity among Saudi adolescents is recommended.

## Data Availability

The raw data supporting the conclusions of this article will be made available by the authors, without undue reservation.
